# Spatio-temporal regulation of circular RNA expression during porcine embryonic brain development

**DOI:** 10.1186/s13059-015-0801-3

**Published:** 2015-11-05

**Authors:** Morten T. Venø, Thomas B. Hansen, Susanne T. Venø, Bettina H. Clausen, Manuela Grebing, Bente Finsen, Ida E. Holm, Jørgen Kjems

**Affiliations:** Department of Molecular Biology and Genetics, Interdisciplinary Nanoscience Center (iNANO), Aarhus University, Aarhus, Denmark; Neurobiology Research, Institute of Molecular Medicine, University of Southern Denmark, Odense, Denmark; Laboratory for Experimental Neuropathology, Department of Pathology, Randers Hospital, Randers, Denmark

**Keywords:** Circular RNA, circRNA, *Sus scrofa*/pig brain, Embryonic/fetal development, Cortical development, CSPP1, Centrosome and spindle pole associated protein 1, HDAC2, Histone deacetylase 2, RIMS2, RIM2, Regulating synaptic membrane exocytosis 2, TLK1, Tousled-like kinase 1, TMEFF1, Transmembrane protein with EGF-like and two follistatin-like domains 1, NDFIP2, Nedd4 family interacting protein 2, ISH, in situ hybridization

## Abstract

**Background:**

Recently, thousands of circular RNAs (circRNAs) have been discovered in various tissues and cell types from human, mouse, fruit fly and nematodes. However, expression of circRNAs across mammalian brain development has never been examined.

**Results:**

Here we profile the expression of circRNA in five brain tissues at up to six time-points during fetal porcine development, constituting the first report of circRNA in the brain development of a large animal. An unbiased analysis reveals a highly complex regulation pattern of thousands of circular RNAs, with a distinct spatio-temporal expression profile. The amount and complexity of circRNA expression was most pronounced in cortex at day 60 of gestation. At this time-point we find 4634 unique circRNAs expressed from 2195 genes out of a total of 13,854 expressed genes. Approximately 20 % of the porcine splice sites involved in circRNA production are functionally conserved between mouse and human. Furthermore, we observe that “hot-spot” genes produce multiple circRNA isoforms, which are often differentially expressed across porcine brain development. A global comparison of porcine circRNAs reveals that introns flanking circularized exons are longer than average and more frequently contain proximal complementary SINEs, which potentially can facilitate base pairing between the flanking introns. Finally, we report the first use of RNase R treatment in combination with in situ hybridization to show dynamic subcellular localization of circRNA during development.

**Conclusions:**

These data demonstrate that circRNAs are highly abundant and dynamically expressed in a spatio-temporal manner in porcine fetal brain, suggesting important functions during mammalian brain development.

**Electronic supplementary material:**

The online version of this article (doi:10.1186/s13059-015-0801-3) contains supplementary material, which is available to authorized users.

## Background

Recently, the phenomenon of circular RNA (circRNA) has gone from being perceived as a rare curiosity to having a central regulatory role in RNA metabolism [1–4]. By adding a new layer of complexity to RNA biology, circRNA may be an integral regulatory entity required to develop and maintain multiple distinct mammalian cell types and organs from the same genetic information. In *Drosophila*, neural levels of circRNAs were found to increase throughout life, which may suggest an active role of circRNAs in maintenance of the aging brain [5]. Even more complex regulation would be expected to take place during prenatal brain development, as correct neuronal architecture is highly dependent on proper timing of cell division, migration and differentiation. Here we address the potential functional role of circRNAs in prenatal brain development. Specifically, we have quantified the spatio-temporal prevalence of circRNA levels during development in the fetal mammalian brain by Illumina deep sequencing of porcine brain samples.

Current knowledge of cellular and molecular mechanisms in brain development has so far mainly been obtained from studies on the rodent smooth-surfaced lissencephalic brain [6]. Across mammalian evolution the relative size of the human brain has increased, which has been facilitated mainly through gyration, a progressive expansion and convolution of the cortical surface [7]. Aberrant regulation of gyration is connected to a significant proportion of mental retardation disorders and epilepsy in children [8]. Consequently, to investigate the distinctive features of the gyrencephalic brain and implications for human disease, alternative animal models to rodents are needed. The domestic pig (*Sus scrofa*) is increasingly being used as a model system for humans in biomedical research spanning neuroscience, cardiovascular and metabolic disease and even for xenotransplantation [9, 10]. The porcine brain is comparable to the human brain based on anatomy, histology, growth and development, and its size enables further and earlier dissection compared with rodents [9]. Furthermore, a high quality draft pig genome has recently become available [11]. Hence, the pig is an appropriate non-primate model for investigating mechanisms of fine-tuned regulation needed in proper brain development.

Brain development is known to be intricately controlled by various noncoding RNAs such as microRNAs and long non-coding RNAs [12, 13]. Recently, considerable attention has been turned to the circularization of exonic sequences, a process known as back-splicing. Even though a limited number of exonic circRNA species have been known for several years, such as the circular testis‐determining RNA *SRY* [1], the scope of circRNA production in mammalian cells has only recently been appreciated. The newly discovered circRNA sponge for miR-7 (CiRS-7) was found to be a potent sponge for cellular miR-7, causing reduction in the active miR-7 pool [2, 4]. Also, it was recently established that the biogenesis of a circRNA derived from the muscleblind (*MBL1*) locus in *Drosophila* was stimulated by the Mbl1 protein, thereby reducing *MBL1* mRNA production. The MBL1 circRNA was shown to interact with Mbl1 and potentially function as a decoy producing an autoregulatory loop that ensures controlled expression of Mbl1 [14].

The specific mechanism underlying circRNA biogenesis has not yet been completely elucidated, although a stimulatory effect from complementary ALU elements in the introns flanking the circularized exons was reported [15]. We and others have further investigated how complementary base pairing sequences in each flanking intron can stimulate biogenesis [2, 16–19], in line with earlier investigations on the *SRY* gene [1]. However, not all circRNAs have such flanking complementary sequences and often exons are flanked by complementary sequences without resulting in circRNA formation. While complementary ALU elements and other base pairing sequences contribute to circRNA production and are significantly associated with circRNA-producing loci, this only explains the biogenesis of a subset of circRNAs. One confounding effect is that base pairing ability within introns counteracts the circRNA promoting effects of complementary sequences in introns across exons [17].

The use of ribosomal RNA (rRNA) depletion instead of poly(A) purification prior to RNA sequencing has enabled the analysis of non-polyadenylated RNA species, including circRNAs. We have utilized rRNA depletion followed by Illumina sequencing to investigate the circRNA content at six different time-points during fetal pig brain development in the cortex of this gyrencephalic brain. Samples from four other embryonic brain regions were also sequenced and investigated, yielding a comprehensive spatio-temporal map of circRNA expression in fetal mammalian brain.

## Results

To picture the circRNA landscape during the course of mammalian embryonic brain development we adopted a deep sequencing-based approach for circRNA detection and applied it to pig (*S. scrofa*) brain samples ranging from very early development at embryonic day (E)23 until time of birth (E115). To ensure an unbiased representation of linear and circRNA we refrained from doing both poly(A) selection and RNase R treatment to remove linear RNA. Total RNA was depleted of rRNA prior to library preparation and sequenced using the paired-end (2 × 100 nucleotide) Illumina technique. Data were processed to remove adapter sequence and low quality sequence information. Initially, we identified circRNA in early embryonic forebrain tissue (E23) and cortex samples (E42, E60, E80, E100 and E115) using the Memczak et al. pipeline [4] with default settings. At E23 the porcine brain is too under-developed to allow fine dissection so the entire forebrain is used as representative for cortex at E23. In total, we identified 9377 circRNA candidates with a minimum of two reads spanning the back-splice junction, using the standard filter settings suggested by the authors. To investigate potential erroneous circRNA detection derived from mis-annotation of linear transcripts, we added an extra step to the circRNA annotation pipeline where all candidate circRNA splice junctions were mapped to the porcine genome using the BLAT tool [20]. Junctions that mapped to the genome in a linear manner were deemed as wrongly annotated circRNA. This extra step showed that 1.8 % of candidate circRNAs were likely to be mis-annotated linear transcripts, typically exhibiting repetitive exonic sequence elements or derived from neighboring genes with high homology. Notably, 10 of the 50 highest expressed circRNAs detected were found to be such mis-annotations. To prevent mis-annotations of circRNAs we raised the filtering requirements from the suggested mapping quality of 35 at one circRNA terminus to a more conservative mapping quality of 40 (maximum score), corresponding to a requirement for an exact match to both termini of the circularized exons. This reduced the total set of detected candidate circRNAs to 5585 (Additional file 1). To allow direct comparison between samples, circRNAs in each sample were normalized as number of back-spliced reads per million raw reads (RPM). A minimum RPM cutoff was set to 0.05, which requires more than one back-spliced read for the sample with the lowest number of raw reads (21 million raw reads in the cortex at E60). At these settings 4634 circRNAs are expressed above 0.05 RPM in at least one cortex sample (Table 1).

**Table 1:** CircRNA expression in porcine cortex

	E23	E42	E60	E80	E100	E115	All
Number of circRNAs	1511	2604	2681	1494	1091	945	4634
Genes producing linear transcripts	12,244	11,536	11,117	11,795	11,732	11,350	13,854
Genes producing circRNAs	1012	1462	1545	1012	774	658	2195
Percentage of genes producing circRNAs	8.3 %	12.7 %	13.9 %	8.6 %	6.6 %	5.8 %	15.8 %
CircRNAs expressed above host	45	48	94	37	44	38	138
Genes producing three or more circRNAs	107	259	234	91	71	56	365
Percentage of genes producing three or more circRNAs	0.87 %	2.25 %	2.10 %	0.77 %	0.61 %	0.49 %	2.63 %

CircRNAs expressed in porcine cortex from E23 until E115. Only circRNAs with expression above 0.05 back-spliced reads per million raw reads (RPM) are included. Annotated gene expression cutoff is set at 1 FKPM (fragments per kilobase of exon per million fragments mapped)

The expression level of linear transcripts was determined as “fragments per kilobase of exon per million fragments mapped” (FPKM) and an FPKM score of 1.0 in at least one sample was chosen as a minimum expression cutoff for linear transcripts.

**Fig. 1:**
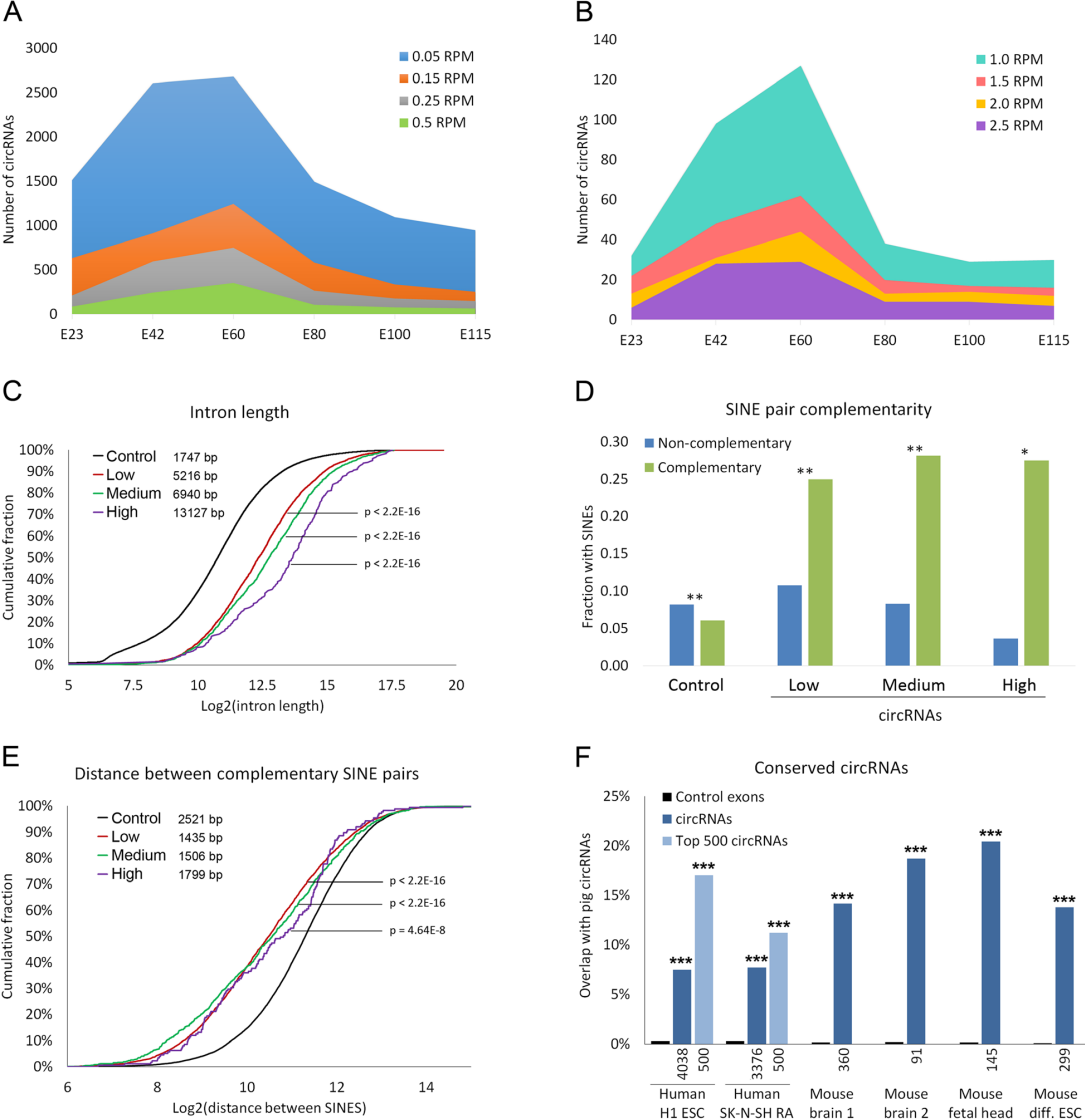
Features of cortical circRNAs. **a**, **b** The number of circRNAs expressed at various cutoff expression levels. **c** Cumulative plot showing length of introns flanking circRNAs with expression levels categorized as either low (0.05 to 0.5 RPM, *red line*), medium (0.5 to 2.5 RPM, *green line*) or high (>2.5 RPM, *purple line*) compared with introns flanking exclusively linear spliced control exons (non-circRNA-forming internal exons from genes that do form circRNAs at other exons, *black line*). Median intron lengths are shown. Introns of all three circRNA subgroups are significantly larger than the control. **d** The intron groups from (**c**) examined for non-complementary and complementary short interspersed nuclear elements (SINEs) within the first 500 bp of flanking introns. **e** A cumulative plot of the distance between complementary SINE pairs in flanking introns of the intron groups from (**c**). The distance between flanking SINEs is the total genomic distance minus the distance between splice sites involved in circularization. The median distances between complementary SINE pairs are shown. **f** The percentage of human and mouse circRNAs with identical counterparts in embryonic pig cortex after use of UCSC liftOver tools (*blue*) compared with random in silico-generated control circRNAs (*black*). *P* values: *P < 10^−5^, **P < 10^−10^, ***P < 10^−20^. *ESC* embryonic stem cell, *SK-N-SH RA* SK-N-SH neuroblastoma cells differentiated by retinoic acid

Depending on the time-point, circRNAs are detected in 5.8–13.9 % of all expressed genes (Table 1). It should be noted that since this de novo circRNA detection is done without prior knowledge of annotated exons, some circRNAs fall outside the genomic region of known genes (206 circRNAs). Also, several genes produce multiple circRNAs (see below). The numbers of circRNAs produced increased significantly from E23 to E42 and peaked at E60. At E80 the number of expressed circRNAs declined drastically with continuing reduction through E100 and E115. This general pattern is observed for both lowly and highly expressed circRNAs (Fig. 1a, b, respectively). This observation hints that circRNAs have particular widespread functions in the first half of the porcine gestation period.

Based on our cortex dataset, we investigated correlative features associated with a high propensity to form circRNAs. The most pronounced characteristic is that porcine circRNAs more often are flanked by large introns in their host genes compared with the linearly spliced exons (Fig. 1c). Also, circRNAs are more often flanked by introns containing complementary SINEs close to the borders of circularized exons compared with their linear counterpart (Fig. 1d, e). However, introns with proximal flanking SINEs in a non-complementary orientation are not indicative of circRNA formation (Fig. 1d; Additional file 2a). We observe a linear correlation between intron length and distance between complementary intronic SINEs for circRNAs, which is not observed for non-circRNA-flanking introns (Additional file 2b). Thus, the short circRNA-flanking introns have a high propensity to contain proximal complementary SINEs (Additional file 2c), suggesting that SINE-mediated circularization is primarily playing a role in the biogenesis of circRNAs with short flanking introns. This observation seems not to be an inherent link between intron length and SINE distribution, as only a very small difference between SINE distribution in long and short flanking introns is seen in the non-circRNA-producing host gene exons (Additional file 2c).

To elucidate the functional importance of back-splicing we investigated the conservation of gene loci connected with circRNA production in pig reported in this study with previously published datasets from human and mouse rRNA-depleted RNA-seq samples [21–23]. The human data derive from an embryonic stem cell line (H1 ESC) and retinoic acid-differentiated SK-N-SH cells, a neuroblastoma cell line that has undergone neuronal differentiation [23]. The mouse datasets are derived from adult mouse brain [22], mouse fetal head, which primarily contains fetal brain, and retinoic acid differentiated embryonic stem cells [21]. Although circRNAs detected in these samples are available on circBase [24], we reanalyzed the datasets with our more stringent filtering criteria in order to directly compare the data with pig. Conserved circRNA back-splicing was examined by use of the liftOver tool from the UCSC genome browser [25]. In total, 88 % of the detected porcine circRNA splicing regions aligned with the mouse genome. Impressively, 20.4 % of the splice sites involved in circularization events in mouse fetal head were identical to circRNAs in pig. This is a highly significant result when compared with random in silico-generated control circRNAs (Fig. 1f). The human datasets yielded many more circRNAs than the mouse datasets, which is likely to be due to the greater sequence depth for the human samples. Therefore, the overlap between pig and human circRNAs is shown for both the total number of human circRNAs and for the top 500 expressed human circRNAs (Fig. 1f). The most highly expressed circRNAs exhibited a larger degree of conservation than average for the complete set, indicating that these circRNAs are likely to be functionally more important. As a whole, this shows that a large number of back-splicing events giving rise to circRNAs are conserved between pig, mouse and humans.

**Fig. 2:**
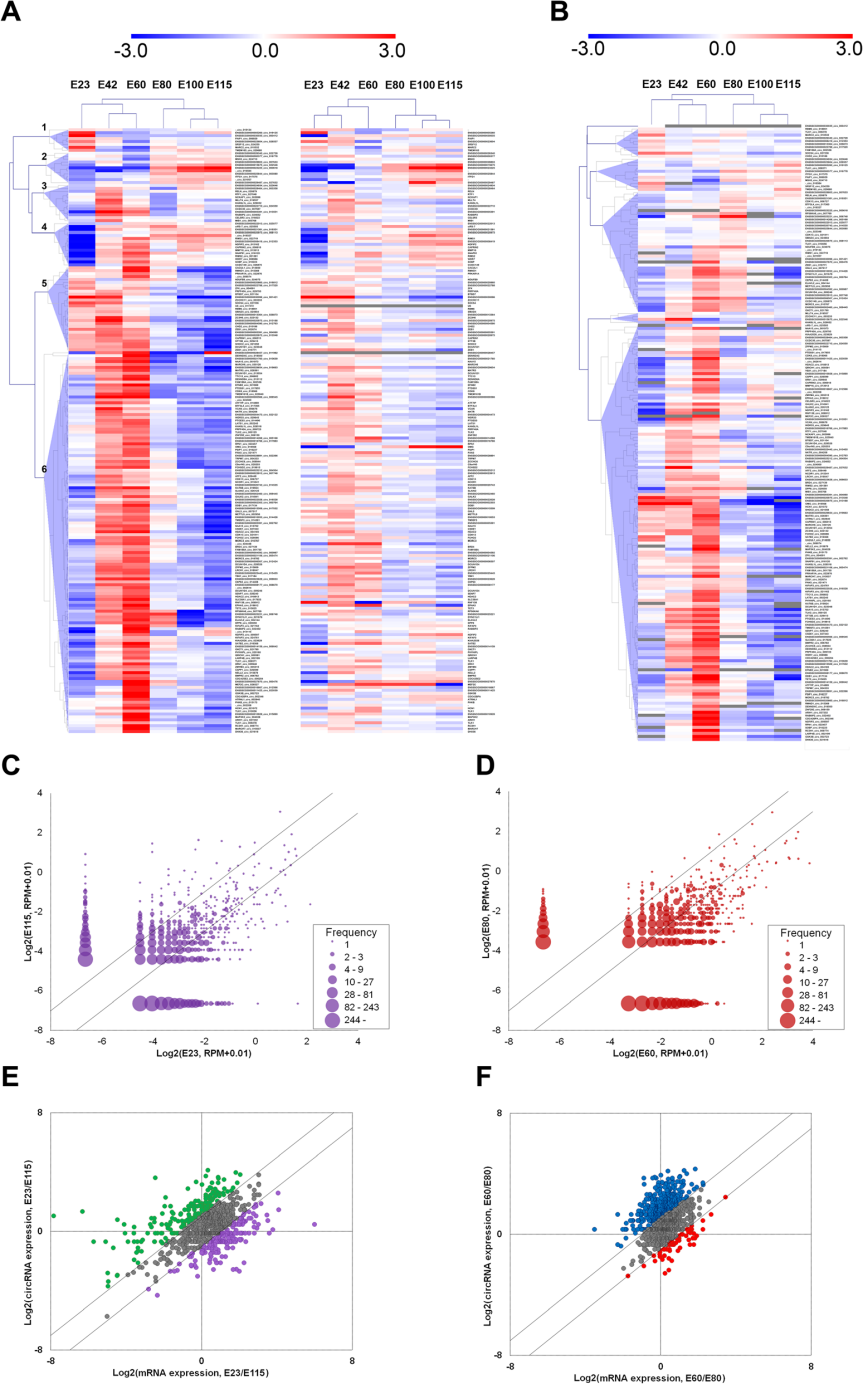
Temporal expression of circRNAs in cortex. **a** Clustered heatmap showing expression patterns of the most highly expressed circRNAs (*left*) and corresponding linear host transcripts (*right*) in matching order. The Pearson correlation coefficient between circRNAs and linear hosts is 0.52. CircRNA clusters with similar expression patterns are numbered and described in the text. **b** CircRNA relative to host gene expression for the highest expressed circRNAs. This shows changes in circRNA expression level independent of the host gene expression. *Grey tiles* indicate ratios which were not calculated due to absent expression of one of the species. CircRNAs with undetected hosts are omitted. **c**, **d** Expression of circRNAs in embryonic cortex at E23 versus E115 (**c**) and E60 versus E80 (**d**). *Diagonal lines* indicate twofold up- and down-regulation. **e**, **f** Analysis of the impact of mRNA host gene expression change on circRNA expression change in embryonic cortex at E23 versus E115 (**e**) and E60 versus E80 (**f**). *Diagonal lines* indicate twofold up- and down-regulation. CircRNA/host expression changes that differ by less than twofold are shown in *gray*. For the other circRNAs (*colored*), expression levels change independently of host mRNAs

To further address the potential functions of circRNA, they were clustered based on their expression profiles. Several circRNAs exhibited similar expression patterns (Fig. 2a, left). Small but distinct groups of circRNAs were expressed either early or late in development (groups 5 and 2, respectively) or predominately at one specific time-point, either E23, E42 or E60 (groups 1, 3 and 4, respectively). A much larger group was expressed mainly at E42 and E60 (group 6). Pairwise comparison of circRNA expression during cortical development reveals that, from E23 to E115, many circRNAs exhibit expression changes, both up- and down-regulated, over this extended period (Fig. 2c). However, when focusing on the limited, but highly biologically relevant, period from E60 to E80 (Fig. 2d) we observe a clear propensity for high circRNA expression at E60. In fact, we find that 94 circRNAs are expressed to a higher extent than their linear counterpart at E60, which is almost twice as many as at any other time-point (Table 1). In total, 138 circRNAs are expressed more highly than their linear counterpart at at least one time-point (Table 1).

The expression profile of circRNAs is expected to be influenced greatly by host gene expression. To address potential regulation of circRNA biogenesis uncoupled from overall expression of host genes, we investigated correlation between circRNAs and host gene linear expression (Fig. 2a, right). Only a moderate Pearson correlation coefficient of 0.52 was found, indicating that other factors influence the propensity to produce circRNAs. To visualize this more directly we calculated the ratio between circRNAs and host genes (Fig. 2b). This showed that, even when correcting for host expression, relative circRNA expression remained highly up-regulated in cortex at E60 for most circRNA genes. When directly comparing mRNA expression changes with circRNA expression changes between cortical time-points, it again becomes evident that altered mRNA expression cannot sufficiently explain the observed circRNA expression. Between E23 and E115, large changes in mRNA expression are observed with low impact on the associated circRNAs (Fig. 2e), whereas from E60 to E80 many mRNAs show modest expression changes but with considerably higher circRNA expression at E60 (Fig. 2f).

Under the assumption that circRNA function will be related to the known function of the host gene, we performed a pathway analysis to predict potential functions of circRNAs up-regulated at E60. This analysis shows a significant contribution to Wnt signaling and axon guidance and, to a lesser extent, to the transforming growth factor (TGF)-beta signaling pathway (Table 2). To examine whether these pathways could be highly associated with circRNA production mainly due to high gene expression, we performed pathway analysis on all highly expressed mRNAs (>50 FPKM) across all cortical time-points, showing axon guidance as the most significant hit and Wnt signaling as the 18^th^ most significant hit (Additional file 3a). This suggests that the top circRNA host gene-associated pathway, Wnt signaling, is not detected due to generally high gene expression. To search for general features of the overrepresented pathways, which might facilitate high circRNA production, we searched all the associated genes for gene length, intron length and number of SINEs (Additional file 3b–d). This showed that axon guidance and Wnt signaling genes are, on average, significantly longer and have a slightly larger proportion of intronic sequence than genes in general. Surprisingly, axon guidance genes have significantly fewer SINEs per gene length.

**Table 2:** Pathway analysis

Term	*P* value	Benjamini q value
Wnt signaling pathway	5.80E-05	0.006
Axon guidance	6.50E-04	0.034
TGF-beta signaling pathway	3.90E-03	0.13

Top three over-represented KEGG (Kyoto Encyclopedia of Genes and Genomes) pathways detected with DAVID for host genes of circRNAs that are expressed above 0.15 RPM at E60 and which show at least a twofold decrease at E80

**Fig. 3:**
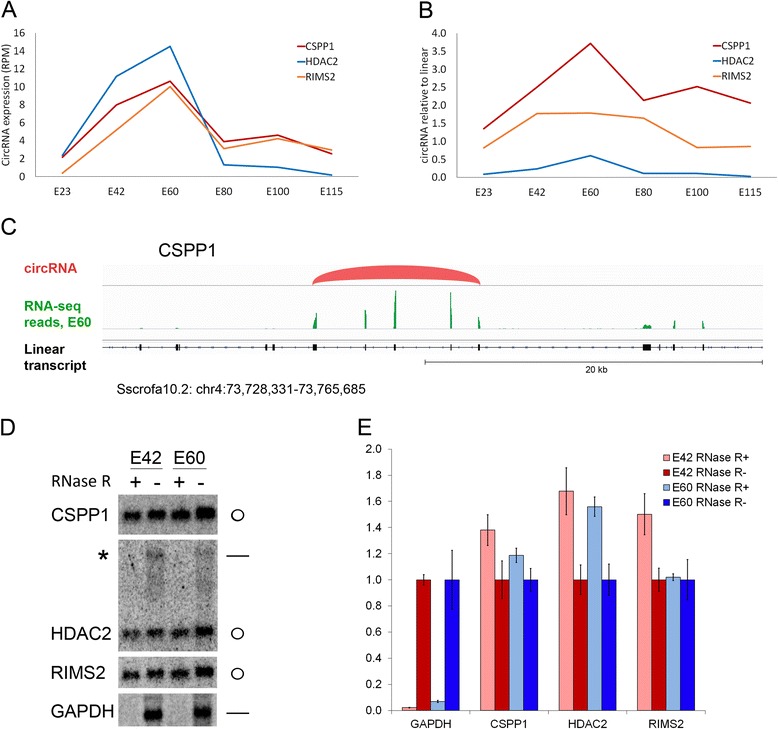
Highly expressed circRNAs. **a** Expression pattern of CSPP1, HDAC2 and RIMS2 circRNAs. **b** Expression relative to linear host expression for CSPP1, HDAC2 and RIMS2 circRNAs. **c** Genome browser view of CSSP1 circRNA (*red*). TopHat2-mapped RNA-seq reads from cortex at E60 are shown in *green*. Intron–exon structure of the CSSP1 gene region is shown below (*black*). Exons contained in the CSSP1 circRNA have larger read density than other exons in the CSSP1 gene. Note that TopHat2 is not able to correctly map back-spliced sequences, so the outermost exonic sequence of a circRNA will appear to have lower coverage than the internal exonic sequences. **d** Northern blots showing RNase R-resistant CSSP1, HDAC2 and RIMS2 circRNAs in cortex at E42 and E60 with linear GAPDH transcript as RNase R-sensitive control. A faint RNase R-sensitive band corresponding to the HDAC2 linear transcript is detected with northern blotting (*asterisk*). Uncropped northern blot lanes are shown in Additional file 4. **e** Quantitative PCR across the back-splice junction of circRNAs from the same RNA samples used in panel (**d**). Error bars represent standard deviation

Three of the most highly expressed circRNAs, expressed from host genes encoding Centrosome and spindle pole associated protein 1 (CSPP1), Histone deacetylase 2 (HDAC2) and Regulating synaptic membrane exocytosis protein 2 (RIMS2), were examined in greater detail. All three circRNAs show peak expression in cortex at E60 (Fig. 3a). In particular, expression levels of CSPP1 circRNA, containing exons 6–10, were much higher than those of the linear transcript from the CSPP1 gene (3.7-fold higher circRNA expression at E60; Fig. 3b). This high relative circRNA expression is also directly evident when viewing the region in a genome browser (Fig. 3c), showing high coverage of the five exons included in the circRNA compared with the surrounding exons. The circular nature of CSPP1, HDAC2 and RIMS2 circRNAs was confirmed by their resistance to RNase R digestion using northern blot and quantitative PCR at the time-points of highest circRNA expression, E42 and E60 in cortex (Fig. 3d, e; uncropped northern blots are shown in Additional file 4). Probes used for northern blotting target both linear and circRNA species, and for HDAC2 an RNase R-sensitive band originating from the linear HDAC2 transcript is visible (asterisk in Fig. 3d). Linear transcripts were not apparent using CSPP1 and RIMS2 probes, reflecting the higher circRNA to linear host ratios observed for these two circRNA species (Fig. 3b).

**Fig. 4:**
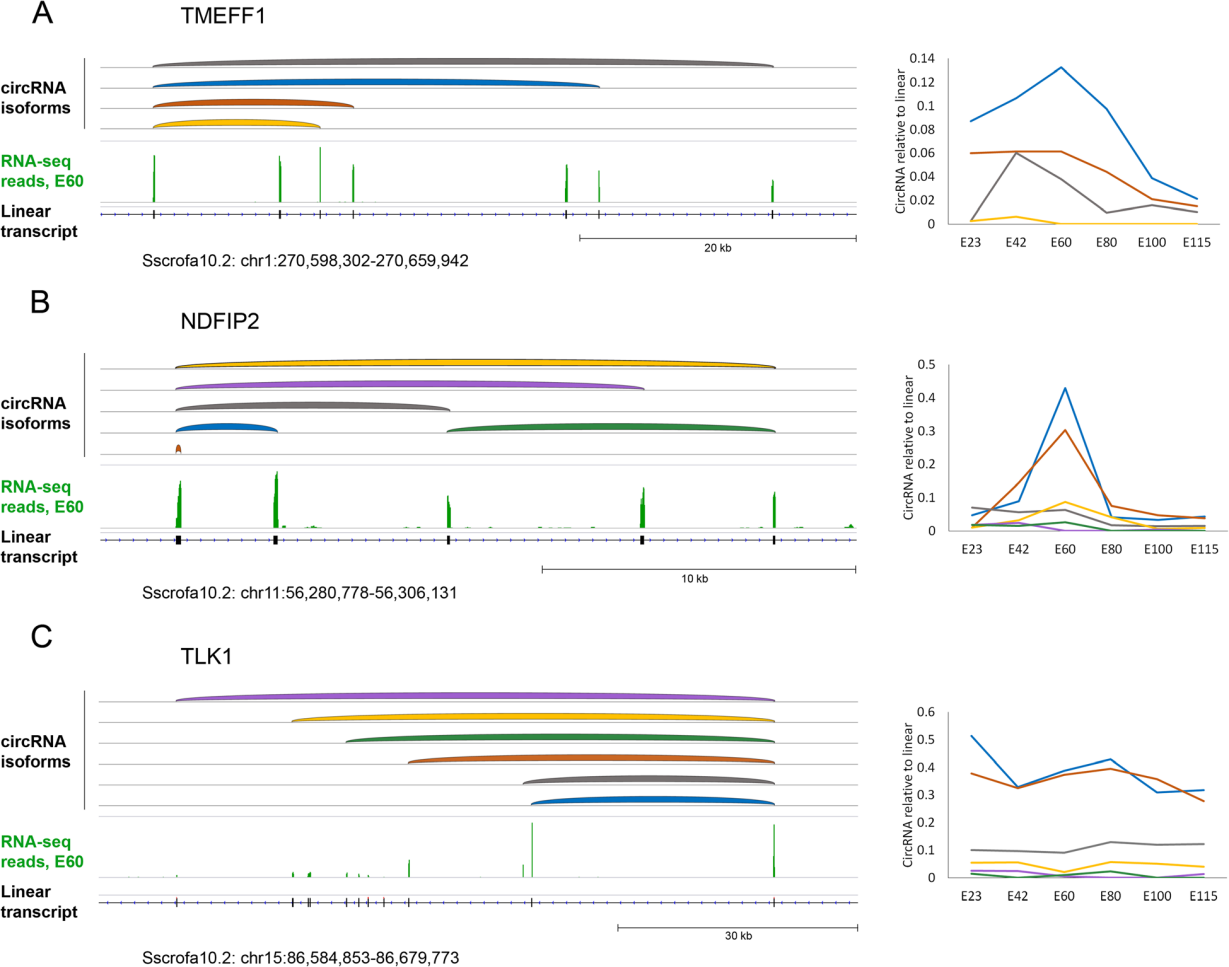
Hot-spot circRNA generation. circRNAs are shown in genome browser views on the left side for TMEFF1 (**a**), NDFIP2 (**b**) and TLK1 (**c**). CircRNA expression levels relative to linear host gene expression levels are shown to the right. The individual circRNA splicing variants are color-coded on the maps (*left*) and expression graphs (*right*). Color denotes the relative circRNA isoform expression. From highest to lowest expressed are: Blue, orange, grey, yellow, purple, green

Many genes produce multiple circRNAs (Table 1). Such circRNA “hot-spot” genes are of particular interest, both with respect to studying the underlining mechanism of circRNAs biogenesis and for elucidating differential regulation of related circRNAs. Indeed, different circRNAs originating from the same host gene can be seen to display dissimilar expression profiles (Fig. 4). RT-PCR was used to validate the existence of multiple different circRNA isoforms from TMEFF1, NDFIP2 and TLK1 host genes (Additional file 5). The predominant circRNA expressed from the TMEFF1 gene peaks at E60 (blue in Fig. 4a), the second highest expressed isoform (orange) remains high steadily through the first half of gestation, whereas the third highest expressed isoform (grey) peaks at E42. Similarly, two of the circRNAs produced from the NDFIP2 gene (blue and orange in Fig. 4b) exhibit a sharp relative expression peak at E60, while other NDFIP2 circRNAs, using the same splice acceptor site, do not (Fig. 4b). Finally, there are many examples of hot-spot circRNAs being expressed at constant levels compared with their linear counterparts, as exemplified by the TLK1 gene (Fig. 4c). Interestingly, most circRNAs from these hot-spot genes involve splicing with one specific exon, either at the splice donor or splice acceptor. This indicates that a strong driver of circRNA splicing resides in the vicinity of these particular splice sites, either in the exons or adjacent introns. Notably, hot-spot splice sites involved in the biogenesis of at least two different circRNA isoforms are, in general, flanked by longer introns than splice sites involved in the biogenesis of a single circRNA isoform only (Additional file 2d).

**Fig. 5:**
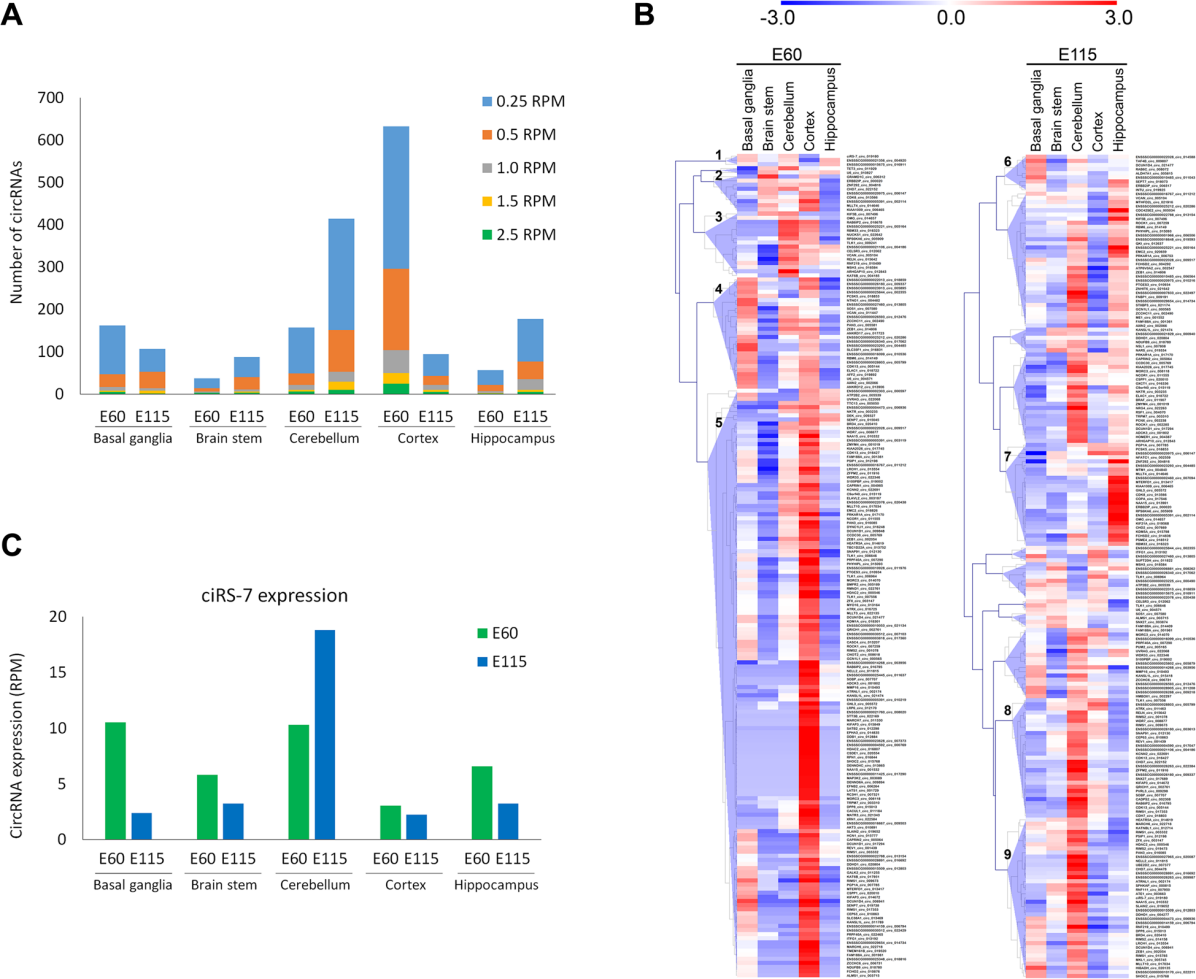
CircRNA expression in various embryonic brain regions. **a** The number of circRNAs expressed at various cutoff expression levels in various tissues. **b** Clustered heatmaps showing expression patterns of the highest expressed circRNAs at E60 (*left*) and E115 (*right*) tissues. **c** Normalized expression levels of CiRS-7 at E60 and E115 in indicated tissues

To gain insight into the circRNA landscape in other brain regions, tissue from basal ganglia, brain stem, cerebellum and hippocampus, obtained from E60 and E115 fetuses, were analyzed for circRNA expression. This allowed evaluation of the spatio-temporal circRNA expression patterns from mid-gestation until time of birth. The expression of circRNAs varied dramatically between the tissues, being highest in cortex and cerebellum and lowest in brain stem (Fig. 5a). The timing of circRNA expression also exhibited different trends. In contrast to cortex where circRNA expression peaked at E60, cerebellum, brain stem and hippocampus circRNA were most predominant in new-born pigs (E115; Fig. 5a). This indicates a particular need for circRNAs at different time-points in different tissues.

To identify tissue-specific circRNA patterns, clustered heatmaps were generated for all E60 and E115 samples, respectively (Fig. 5b). These revealed large spatial differences in expression patterns: at E60 we generally observed low expression in brain stem, except from one small group of brain stem-specific circRNAs (group 2). Distinct tissue-specific clusters of circRNAs are highly expressed in cerebellum (group 3) and basal ganglia (group 4). The large cluster of circRNAs found is in cortex and appears to be cortex-specific (group 5). A small subset of circRNAs is particularly lowly expressed in cortex at E60 (group 1). Interestingly, the highly expressed and well-studied circRNA CiRS-7 is among these. Also, at E115, distinct groups of circRNAs show specific spatial expression, such as in basal ganglia (group 6), hippocampus (group 7) and cerebellum (groups 8 and 9). At E115, CiRS-7 is the most highly expressed circRNA in cerebellum with an expression of 19 RPM (Fig. 5c), which is in agreement with our previous study on CiRS-7 expression [26]. In other tissues CiRS-7 is most highly expressed at E60, showing that circRNA expression can vary greatly in a spatio-temporal manner (Fig. 5c), indicating that a particular circRNA may exert its role at different time-points in different brain tissues.

**Fig. 6:**
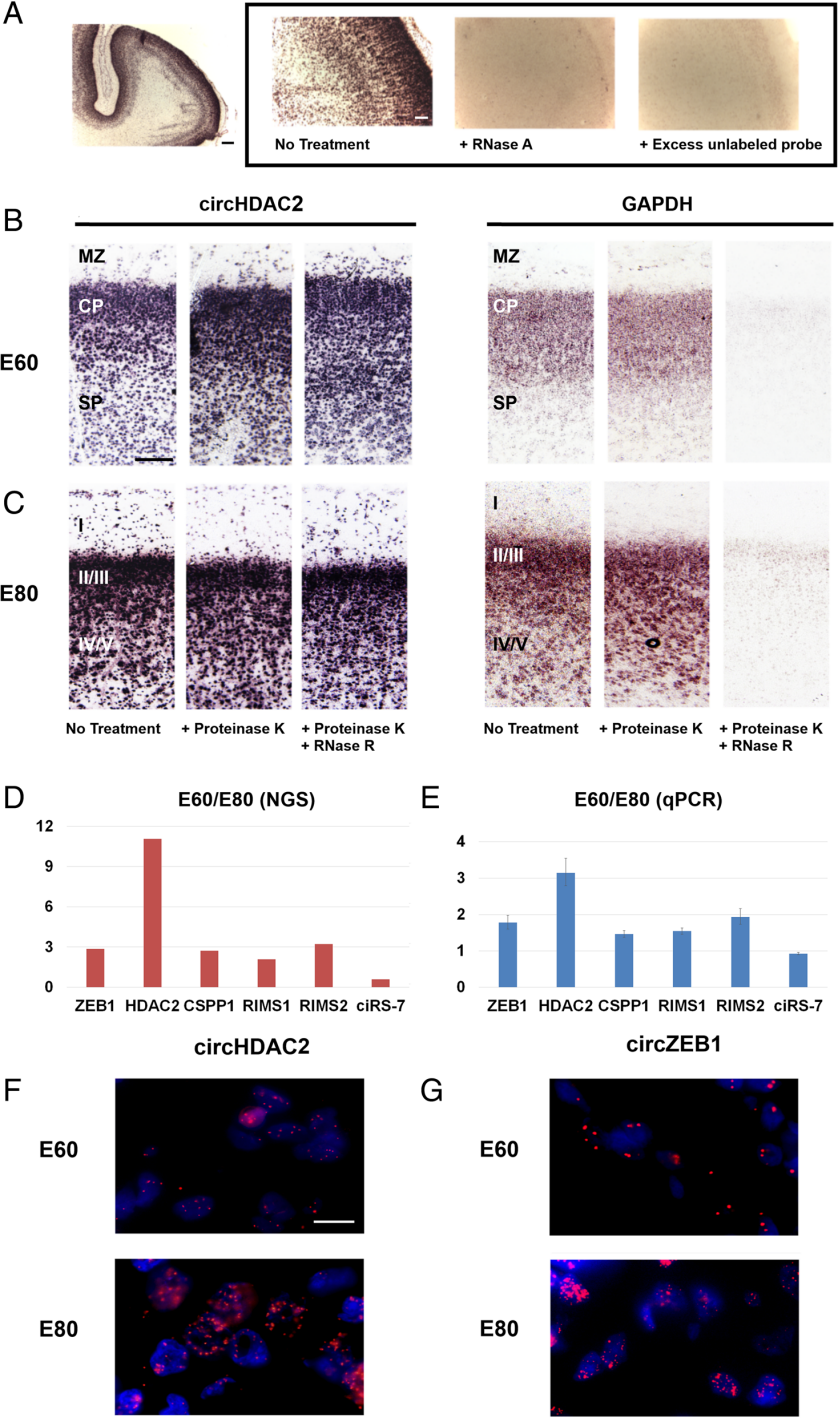
In situ hybridization (ISH) and quantitative PCR validation of circRNA in pig embryonic brain. **a** Localization of circHDAC2 at E80. The circular isoform was detected by in situ hybridization using a 20-nucleotide alkaline-phosphatase conjugated LNA probe matching the back-splice junction (10 nucleotides on each side). Standard controls were conducted applying either RNase A or 100-fold excess of unlabeled probe. **b**, **c** Distribution of circHDAC2 in cortex. Detection of circHDAC2 or GAPDH (linear control) as described in (**a**) at E60 and E80, respectively. Treatment with proteinase K followed by RNase R reduced the GAPDH signal while not affecting circHDAC2, supporting the circular nature of the probe target. Proteinase K alone did not reduce either signal. Roman numerals indicate cortical layers. **d**, **e** Change in circRNA levels between E60 and E80. E60/E80 ratios for six different circRNAs, according to Illumina next generation sequencing (*NGS*) data (one sample) (**d**) and quantitative PCR (*qPCR*) on tissue used for ISH (two biological replicates, each done in triplicate) (**e**). **f**, **g** Subcellular localization of circHDAC2 (**f**) or circZEB1 (**g**) in the subplate at E60 and in layer IV/V at E80. Subcellular localization was visualized using panomics probes for high-resolution ISH and DAPI for nuclear localization. *CP* cortical plate, *MZ* marginal zone, *SP* subplate. Scale bars: 200 μm (**a**), 50 μm (**b**, **c**), and 10 μm (**f**, **g**)

The most highly expressed circRNA at E60 is circHDAC2, which accordingly was chosen for further investigation of the spatial distribution using in situ hybridization with an alkaline phosphatase-coupled LNA probe spanning the back-splice junction of circHDAC2. With this approach, the presence of the circHDAC2 in embryonic porcine brain tissue could be directly visualized (Fig. 6). Pre-treatment of the tissue with either RNase A or excess unlabeled oligo as competitor completely abolished the signal (Fig. 6a), indicating that the signal was specific to the splice junction and not a general absorption artifact. To further investigate whether the targeted RNA was indeed circular, the tissue was pretreated with RNase R to remove linear RNA species. A successive treatment of the tissue with proteinase K and RNase R led to removal of the linear mRNA for GAPDH whereas the signal from the circular circHDAC2 was unaltered (Fig. 6b, c). Together these data demonstrate the potential for detecting circRNA species in situ using structure-dependent RNase enzymes.

Based on the Illumina deep sequencing data, a decrease in the total amount of circHDAC2 was expected from E60 to E80; however, this trend was not obvious from the in situ images. Several factors, such as difference in protein binding and base-pairing to other RNA molecules, may obstruct access of the probe to the back-splice junction during the in situ hybridization procedure. Furthermore, differences in cellular permeability to the probes may also vary at different developmental stages. Therefore, RNA was extracted directly from tissue slices used for in situ hybridization experiments, and circRNA was quantified by quantitative RT-PCR using probes spanning the back-splice junction (Fig. 6e). The relative circHDAC2 expression between E60 and E80 followed the same trend observed in the Illumina sequencing data (Fig. 6d). Consequently, while our experimental in situ detection protocol for circRNA provide a valuable tool for in situ visualization of circRNA, the results should not be regarded as quantitative.

As an alternative visualization method, allowing for a more high-resolution subcellular localization of circHDAC2, the panomics probe system was applied. Both at E60 and E80, the circHDAC2 signal was associated with the nucleus, which contrasts with the previous reported localization of circRNAs (Fig. 6f). Interestingly, when probing against another circRNA, circZEB1, a change in subcellular distribution from E60 to E80 was observed (Fig. 6g). At E60 the circZEB1 signal was detected both with DAPI and in the cytoplasm, whereas at E80 the signal exclusively associated with the nucleus. As seen for the alkaline phosphatase-coupled LNA probes, signal intensity cannot be taken as a quantitative measure for circRNA abundance.

## Discussion

Precisely timed gene expression and alternative splicing patterns are of great importance for the developing nervous system. Back-splicing events significantly increase the complexity of splicing, leading to formation of a large number of specific circRNA species, some of which have important regulatory potential. According to the data presented here, circRNA levels in the porcine brain display complex dynamic changes in a spatio-temporal manner, correlating with tissue-specific events, such as gyration. Hence, alternative circRNA formation may constitute a novel regulatory layer in brain compartmentalization and development. Induction of neural differentiation in established murine and human cell culture models has recently been associated with an increase in circRNA level for the majority of circRNAs examined to date, including circRIMS2 [27]. Also, circRNAs were found to accumulate with age in the head of *Drosophila* [5]. Together, these findings may lead critics to speculate whether circRNAs are a mere by-product of transcription, passively accumulating with age. During embryonic development in the brain, however, many circRNAs show distinct expression patterns uncoupled from their linear counterparts. For example, expression of circCSPP1 was shown to increase from E23, peak at E60 and thereafter, with fluctuations, decrease towards the time of birth (Fig. 3a, b).

The RIMS2 circRNA was recently shown to be highly expressed in human cortex, while in mouse it is almost exclusively located in the cerebellum [27]. We observe a peak of circRIMS2 expression in the developing embryonic pig cortex (Figs. 3 and 6d, e). This indicates that organism-, tissue- and developmental stage-specific regulatory mechanisms are controlling circRNA levels in the brain. We discovered a generally high expression of circRNAs in cortex at early to mid-gestation (E42 and especially E60; Figs. 2 and 5a, b). Interestingly, these time-points correspond to a period of major neurogenesis in the embryonic pig and underscore the potential status of circRNAs as prime candidates for developmental control in embryonic brain.

In accordance with this, pathway analysis of the genes giving rise to the circRNAs peaking at E60 reveals a significant predominance of genes associated with axon guidance, Wnt signaling and, to a lesser extent, the TGF-beta signaling pathway (Table 2). An impact of circRNAs on Wnt signaling, axon guidance and TGF-beta signaling pathways would be of great interest since early and intermediate fetal brain development is characterized by extensive neural differentiation and neuronal migration, processes that are strongly impacted by the three over-represented pathways [28–31]. Specifically, in the period from E60 to E80, where we observe the most extreme shift in circRNA expression patterns, the most dramatic morphological change occurs in the fetal porcine cortex, as neuronal migration causes the smooth (lissencephalic) E60 brain to acquire the gyri and sulci characteristic of the gyrencephalic brain [32]. The TGF-beta signaling pathway, which also has an overrepresentation of circRNAs, plays an important role in specification of axons and dendrites during embryonic brain development and has important neuroprotective functions [31, 33]. TGF-beta signaling also facilitates correct neural migration by controlling radial glia cell differentiation during cortex development [28]. Wnt signaling is important for neuronal progenitor differentiation and formation of neural circuits; the latter through its impact on axon guidance and development of dendrites and axons and through its promotion of synaptogenesis [29, 30]. A potential neural involvement of circRNAs in such fundamental pathways could have an immense impact on correct embryonic brain development, with implications for adult brain function and disease.

Importantly, we clearly see that circRNA expression is often uncoupled from host gene expression. This supports the idea that the circularization process itself is tightly controlled in a manner similar to conventional alternative splicing. This is in agreement with recent publications in the field reporting low correlation between circRNA abundance and expression levels of linear host genes across multiple cell lines [23, 27]. It should be noted that we are measuring the steady state levels of RNA in the tissue samples, which are balanced between RNA transcription and degradation processes. Therefore, elevated circRNA levels relative to host gene linear transcript levels could be caused by either increased back-splicing, increased degradation of the linear transcript or reduced degradation of the circRNA, mechanisms that are indistinguishable.

Based on their high and differentially regulated expression levels and conservation across species, it seems highly likely that circRNAs are involved in development of the mammalian brain, introducing yet another layer of RNA function to this complex organ. Examining the roles of individual circRNAs will be the subject of future investigations. We here investigate three of the most highly expressed circRNAs (CSPP1, HDAC2 and RIMS2) in more detail and validate their presence and circular nature by northern blotting, with and without RNase R treatment. Interestingly, the host genes of these three circRNAs have been shown to be important for brain development or synaptic plasticity [34–38]. Mutations in the CSPP1 gene have been shown to cause a developmental brain disorder called Joubert syndrome, and CSPP1 was found to be involved in neural-specific functions of primary cilia [36]. Inhibition of histone deacetylases by valproic acid has been shown to negatively affect production and differentiation of neural stem cells [35]. HDAC2 has been shown to negatively regulate learning and memory by affecting synaptic plasticity and causing persistent changes in neural circuits [38]. RIMS2, also known as RIM2, is involved in vesicle docking and priming at presynaptic active zones and facilitates Ca^2+^-mediated neurotransmitter release by tethering Ca^2+^ channels to the presynaptic active zones [37, 39].

### Intronic features facilitate circRNA formation

We find that circularized exons more frequently are flanked by large introns and proximal complementary SINEs compared with exclusively linear spliced exons (Fig. 1c–e). This is in agreement with recent reports investigating *Caenorhabditis elegans*, mouse and human circRNAs [15–17, 19]. Longer introns could act to slow the canonical splicing process, allowing time for the circRNA-forming back-splicing event to take place. Also, longer flanking introns would have a greater chance to contain elements promoting base pairing across flanking introns, such as the previously reported complementary ALU repeats [15–17] or the related SINEs reported here. In agreement with Jeck et al. [15] we find that SINEs in introns flanking circular exons are more likely to be complementary. This is inferred from the observation that the ratios between complementary and non-complementary SINE pairs are higher for highly expressed circRNAs (Fig. 1d). The importance of complementary sequences in adjacent introns for circularization was also observed when designing expression vectors for circRNA [26]. Hence, an attractive mechanistic model could be that splice acceptor and donor sites are brought into proximity through base pairing between flanking introns, and this may, in turn, facilitate back-splicing. However, alternative mechanisms for circRNA biogenesis cannot be excluded based on the observation that only approximately half of the observed circRNAs in pigs have complementary SINEs within 1500 bp of the adjacent introns.

### Hot-spot circRNA-generating genes

While only a limited subset of genes produce circRNAs, a fraction of these produce multiple circRNAs. The circRNAs produced from the hot-spot genes often originate from back-splicing events between one particular exon and several others within the same host gene. Strikingly, we find that individual circRNAs from the same host gene can display divergent expression signatures, which demonstrates that some *trans*-acting factor(s) may be responsible for differential expression of circRNAs. The number of genes producing multiple circRNAs is under tight temporal control, with 339 hot-spot genes at E60 and only 49 at E115. In fact, these hot-spot genes account for most of the extra circRNAs observed at E60 relative to the later time-points. This complex nature of circRNA biogenesis raises a number of interesting questions. What regulates the differential appearance of circRNAs produced from the same host gene and do the multiple circRNAs from the same hot-spot genes have distinct functions? The striking resemblance to conventional alternative splicing, where particular splice sites are often spliced to two alternative or more splice sites, suggests that *cis*-acting RNA regulatory elements, bound by transacting protein splicing regulators, are at play. It remains to be seen whether back-splicing can lead to the same level of functional diversity as regular alternative splicing of mRNA.

### *In situ* visualization of circRNA species

To directly visualize circRNA species, we devised a new protocol involving RNase R treatment of fixed tissue samples to remove background caused by linear transcripts. Using this protocol we confirmed the specificity of our probe for back-spliced circHDAC2 (Fig. 6b, c). Our in situ data also revealed an interesting transition of ZEB1 circRNA from being cytoplasmic or perinuclear at E60 to exclusively nuclear at E80 (Fig. 6g). The mechanism behind this developmentally coordinated translocation and the potential switch in function await further investigation.

## Conclusions

For the first time, circRNA expression has been examined in the developing fetal porcine brain. Introns flanking circRNAs were shown to be significantly larger than non-circRNA flanking introns and associated with proximal complementary SINEs. To visualize circRNA in situ, we devised a novel protocol relying on RNase R treatment of fixed tissue to efficiently remove linear RNA species and enrich for circRNAs. Through a spatio-temporal examination, we observed a drastic difference in circRNA expression. In particular, we found a subset of genes that exhibited a highly complex pattern of back-splicing within single genes. A dramatic shift from high circRNA expression observed in cortex at E60 to low expression at E80 indicates that a large group of circRNAs may play roles in the developing mammalian cortex consistent with a high level of conservation between mouse, pig and human.

## Materials and methods

### Sample preparation

All procedures involving animals described in the present study were reviewed and approved by the Danish Experimental Animal Inspectorate (“Rådet for Dyreforsøg”), Danish Ministry of Justice. Dissected porcine fetal brain samples were transferred to dry ice. High molecular weight RNA, above 200 nucleotides, was purified using the MirVana kit (Ambion) and used for Illumina sequencing. For circRNA validations with northern blot analysis and PCR methods total RNA was purified using TRIzol.

### Northern blot analysis

For each fetal pig brain sample, 10 μg of RNA was incubated with or without 10 U RNase R (Epicentre) at 37 °C for 10 min. RNase R-treated RNA was visualized using denaturing agarose northern blotting along with untreated RNA using probes against CSPP1, HDAC2, RIMS2 and GAPDH: CSPP1, 5′- TGG AGT AAA CTG ATG GGG CAG GTG GGA CAG GCG GGG CAG ATA AAG GAG GGA GAG GTG TCT GGA AAG CTA CTC TGG GTC TTT CAG GAG GTA TCA TCT CTT CAA AGT GTC TCG GTG CCA CAC-3′; HDAC2, 5′- CCA AGT CTA TCA CCA GAT AAT GAG TCA GCA CCA CAT TGT AAC ACC ACA GCA CTA GGT TGA TAC ATC TCC ATC ACT TTT GAG ATA ATA GGT TTA AAT ATC TGC CCA TAT GAT TCA TCA TCT-3′; RIMS2, 5′- TTG GCC GTT CTG ATT GGA CAG ACA TGT AGC TTG TGC TGC TGA AAC GAG AAG CAC TAC TAG TCC TTG AAA CCG CAG ATA TAT CAC TTA CAT CAC TGT CCG AAG ATT TAG TGG AAA TAT TAT-3′; GAPDH, 5′- GGA GGC CAT GTG GAC CAT GAG GTC CAC CAC CCT GTT GCT GTA GCC AAA TTC ATT GTC GTA-3′.

### Library preparation and Illumina sequencing

For each sample 4 μg of RNA was treated with DNase using a turbo DNA-free kit (Ambion). RNA was then rRNA depleted using the Ribo-Zero Magnetic Kit (human/mouse/rat; Epicentre). Sequencing libraries were generated using the ScriptSeq v2 kit (Epicentre), quality controlled on the 2100 Bioanalyzer (Agilent). Illumina sequencing was performed at the Beijing Genomics Institute (BGI). One animal was used per time-point. Validation with additional biological replicates was done with quantitative northern blotting, quantitative PCR and RT-PCR.

All Illumina sequencing data have been submitted to the Gene Expression Omnibus (GEO) under accession number [GEO:GSE71832].

### Data analysis

Sequencing data were quality (Phred score 20) and adapter trimmed using Trim Galore. Filtered data were mapped to the porcine genome (Sscrofa10.2/SusScr3) using TopHat2 [40]. Transcript assembly and abundance estimation was performed using Cufflinks [41]. Genome and annotation files were downloaded (ftp://ftp.ensembl.org/pub/release-75). RNA-seq read coverage was visualized for select genes in the Integrative Genomics Viewer [42].

Detection of circRNA was done with find_circ [4], which was downloaded from circBase [24]. The find_circ pipeline was run as suggested by the developers, except for an increased filtering stringency, requiring that both anchor segments map to the genome with mapping scores of 40. Only circRNAs with two or more supporting reads within single samples were kept. CircRNAs were normalized as the number of back-splice junction spanning reads per million raw reads (RPM).

Host genes giving rise to individual circRNAs were identified by matching the genomic location of circRNAs with the location of genes detected by TopHat/Cufflinks using BEDtools [43]. This subsequently allowed comparison of the expression level of each individual circRNA with that of its host, even for currently unannotated porcine genes. FPKM gene expression values estimated by Cufflinks constitute a measure of the host gene expression, normalized according to the total read number and gene length. Prior to direct comparison between circRNA and host gene expression, a length-normalization was implemented for circRNAs. Since longer reads will have more power to detect back-splicing events, read length can be used as length normalization for circRNAs. Our fetal pig brain RNA-seq was done with 100-bp long reads, and to find circRNAs the find_circ software requires anchor sequences of 20 bp on each side of the read, meaning that a back-splicing event can be detected by reads mapping up to 80 bp away in each direction. Thus, a pseudo RPKM expression value for each circRNA was calculated dividing circRNA RPM values by 160 and multiplying by 1000 (to get a value per kilobase), which allowed circRNA-to-host ratios to be calculated.

### Analysis of intron length

Introns were extracted from the Ensembl pig gene annotation file (release 75). Introns flanking exons involved in back-splicing events were further extracted using BEDtools. These were grouped based on the amount of support for the back-splicing event of the circRNA: low (0.05–0.5 RPM), medium (>0.5 to 2.5 RPM) or high (>2.5 RPM). For use as a control, introns from circRNA host genes that are not involved in back-splicing events were likewise extracted. Statistical significance was calculated by two-sided Kolmogorov–Smirnov test.

### Analysis of SINE pair complementarity

Genomic positions of SINEs extracted from the repeat masking track (rmsk) at the UCSC genome browser were intersected with the intron groups used for analysis of intron length using BEDtools to ascertain the intronic distance between complementary and non-complementary SINEs in intron pairs flanking circRNA forming exons or control exons. Statistical significance was calculated by *χ*^2^ test for the bar graph and Kolmogorov–Smirnov test for the cumulative plot.

### CircRNA conservation

Publicly available datasets from human and mouse rRNA depleted RNA-seq samples were downloaded. Human H1 ESC cell line and differentiated SK-N-SH cell line data previously used for circRNA detection [23] were downloaded from the ENCODE project repository [44]. Mouse datasets previously used for circRNA detection [4] were downloaded. The mouse datasets were from adult brain [22], fetal head and differentiated embryonic stem cells [21]. Although these datasets have all previously been used for circRNA detection, we repeated the analysis to ensure consistency with detection method and filtering criteria used for the porcine samples. The human genome (GRCh37/hg19) and mouse genome (GRCm38/mm10) were downloaded from the UCSC genome browser [45].

Conserved circRNA splicing was examined using the liftOver tool from the UCSC genome browser [25]. Presumably due to the low sequence conservation of intronic sequence, simply lifting circRNAs from pig to mouse genome resulted in loss of most multi-exon circRNAs. To prevent this, 20 bp from each end of each circRNA was extracted and lifted to the mouse genome (mm10). CircRNA ends were then recombined and the amount of overlap with circRNAs from other datasets with identical genomic location was counted. With this approach pig circRNAs were lifted from the pig genome (Sscrofa10.2/SusScr3) to the mouse genome (mm10); likewise, human circRNAs were lifted from the human genome (hg19) to mm10. This allows comparison of all circRNAs found in all datasets examined. Also, the UCSC liftOver tool currently only allows lifting of the current pig genome to the mouse genome. To assess statistical significance, in silico-generated control circRNAs were also subjected to this liftOver procedure. The in silico control circRNAs were generated by forming all possible single- and multi-exonic circRNAs from the genes expressed above 10 FPKM, while removing the actual circRNAs detected. In silico control circRNAs were selected at random until reaching the same number of pig circRNAs successfully lifted to the mouse genome (n = 4899). Thereby, it could be assessed whether the amount of pig circRNAs identical to circRNAs from mouse or human were greater than the amount of control matches occurring by chance. Statistical significance was calculated by a *χ*^2^ test.

### Heatmaps

The top 200 expressed circRNAs were log2 transformed, gene mean centered and visualized as heatmaps using the MultiExperiment Viewer (MeV) [46]. Absent expression values were given the lowest score. Where indicated by dendrograms, Pearson correlation average linkage hierarchical clustering was performed.

### Pathway analysis

CircRNAs expressed above 0.15 RPM in E60 that decreased twofold or more in expression at E80 were subjected to KEGG (Kyoto Encyclopedia of Genes and Genomes) pathway analysis using The Database for Annotation, Visualization and Integrated Discovery (DAVID) v6.7 [47, 48] (Table 2). All genes expressed above 1 FPKM in the cortex samples, as measured by TopHat/Cufflinks, were set as the background gene list. All mRNAs expressed above 50 FPKM were likewise subjected to KEGG pathway analysis using DAVID with the same background list.

### In situ hybridization

Fresh frozen pig brains, embryonic stages E60 and E80, were cut sagitally into 10 μm thick sections and placed on RNase free Superfrost plus slides (Thermo Scientific). Sections were stored at -80 °C until further use. Brain samples were obtained from animals different from those used for Illumina sequencing. In situ hybridization was performed as previously described [2, 49] using alkaline phosphatase-labeled DNA and LNA probes recognizing GAPDH mRNA (5′-CCTGCTTCACCACCTTCTTGATGTCA-3′) and circHDAC2 back-splice junction (circHDAC2, 5′-CACCAATATCCTTTGACTGT-3′) (DNA Technology A/S, Denmark). Panomics probes against circHDAC2 and circZEB1 back-splice junctions (5′-TAAACTGAAACTTTAGAGAA-3′) were additionally purchased from Affymetrix (CA, USA) and in situ hybridization performed as described by the QuantiGene® ViewRNA microRNA in situ hybridization guidelines, applying few modifications. Signal specificity was tested as described [49] and RNase R pre-treatment was included as a control for circRNA. Sections were pre-treated with proteinase-K (Sigma-Aldrich; 1 mg/ml) at 37 °C for 1 min prior to RNase R treatment. All circRNA probes were designed to target the 10-nucleotide sequence on each side of the back-splice junction, spanning a total of 20 nucleotides.

### Quantitative RT-PCR

RNA extraction, cDNA synthesis and quantitative PCR were performed as previously described [50]. For each time-point quantitative PCR was done on two biological replicates, each with three technical replicates (Fig. 6), or on one biological replicate with or without RNAse R treatment using four technical replicates (Fig. 4). For each time-point in Fig. 6 one of the biological replicates was from the same animal as was used for in situ hybridization. None of the quantitative PCR tests were run on the RNA used for Illumina sequencing. The following primer sequences were used, all spanning back-splice junctions with sequence specificity checked using BLAST: HDAC2fw, 5′-GGTGCTGACTCATTATCTGGTGAT-3′; HDAC2rev, 5′-CCATCTCTCATTGGAAAATTGACA-3′; ZEB1fw, 5′-TTTCAGTGTTCTTGGAGGTGTGG-3′; ZEB1rev, 5′-TGACTTTTGGATGTTCACGTCTTC-3′; CSPP1fw, 5′-TCAAAAGGAAGATTTGCACGAT-3′; CSPP1rev, 5′-ACCATACTCCATAGAGGGCACAT-3′; RIMS2fw, 5′-GGTAGCTATTGTTGGTCTCTCCC-3′; RIMS2rev, 5′-AATTTTCATTTGGCGATTTCTCT-3′; ciRS-7fw, 5′-TCAGGTCTTCTGGTGTCTACGAT-3′; ciRS-7rev, 5′-TGTTGTTGGAAGACTTGGAATTG-3′; RIMS1fw, 5′-CAAAGTGGTTGCCATAGTGTCTC-3′; RIMS1rev, 5′-AAGTCGATGCACTTTCATTTTCA-3′.

### RT-PCR

The cortex RNA used for Illumina sequencing was also used to validate hot-spot circRNA isoforms. RNA samples were reverse transcribed using M-MLV Reverse Transcriptase (Invitrogen) and 35 cycles of PCR were done with Taq DNA polymerase (Invitrogen). Primers used for divergent PCR were: TMEFF1_FW, 5′- AATGCGCATGTCAGTTTCAG-3′; TMEFF1_RE, 5′-TCAAACCATCTCCGTCTTCTTT-3′; NDFIP2_FW, 5′-TGCTTCTTCAGCATCAGGACT-3′; NDFIP2_RE, 5′-TGCATGGTCTGTGGTACTGG-3′; TLK1_FW, 5′-TCCCACTTGCAACTCCTGTA-3′; TLK1_RE, 5′-CAGTTGCAGTGTTGGAGCTAA-3′.

## Additional files

Additional file 1:
**Spatio-temporal expression of circRNAs.** CircRNAs detected with a minimum of two reads in one or more samples and with a mapping quality of 40 at both termini (anchors). Table 1 shows circular RNAs in whole porcine forebrain at E23 and porcine cortex from E42 until E115. Table 2 shows circRNAs in porcine basal ganglia, brain stem, cerebellum, cortex and hippocampus at E60 and E115. (XLS 4106 kb) Additional file 2:
**Features of cortex circRNAs.** **a** A cumulative plot of the distance between non-complementary SINE pairs in introns flanking circRNAs with expression levels categorized as either low (0.05–0.5 RPM, *red line*), medium (0.5 to 2.5 RPM, *green line*) or high (>2.5 RPM, *purple line*). Introns flanking exclusively linear spliced control exons (non-circRNA forming internal exons from genes that do form circRNAs at other exons) are shown with a *black line*. The figure is related to Fig. 1c. **b** Linear regression shows a positive correlation between intron length and distance between complementary intronic SINEs for circRNAs (*red*). This is not observed for non-circRNA flanking introns (*blue*). **c** Cumulative plot of the distance between complementary SINE pairs for circRNA flanking introns divided into the 50 % shortest introns and the 50 % longest introns (*red* and *orange*, respectively). Short circRNA flanking introns contain more proximal complementary SINEs. This is not observed for introns flanking control exons (*blue* and *green*). **d** Cumulative plot of flanking intron length for hot-spot exons producing two or more circRNAs from either splice acceptor (*red*) or splice donor (*orange*), and exons producing only one circRNA as either splice acceptor (*blue*) or splice donor (*green*). (TIFF 983 kb) Additional file 3:
**Pathway analysis on highly expressed mRNAs and general pathway features.** **a** DAVID pathway analysis was performed on the highest expressed mRNAs in embryonic cortex to find enriched KEGG pathways. mRNAs with expression above 50 FPKM were used for pathway analysis using the same background list as used for pathway analysis of circRNA host gene shown in Table 2. **b**–**d** Genes with two or more exons that are associated with the Wnt signaling pathway, axon guidance and the TGF-beta signaling pathway are examined relative to all genes for gene length (**b**), the proportion of intronic sequence in genes (**c**) and number of SINEs relative to gene length (**d**). Statistical significance for cumulative plots was calculated using the Wilcoxon test. (TIFF 619 kb) Additional file 4:
**Uncropped northern blot lanes.** Northern blot lanes for circRNAs depicted in Fig. 3d are shown uncropped. Ethidium bromide stain of the gel prior to northern blot is also shown. (TIFF 3505 kb) Additional file 5:
**RT-PCR validation of hot-spot circRNA isoforms.** RT-PCR with divergent primers showing multiple circRNA isoforms for the host genes TMEFF1, NDFIP2 and TLK1. Gel images show the sizes of back-spliced amplicons from RT-PCR. On the right, the expected size of circRNAs from RNA-seq is shown, with font size indicating general expression level. (PDF 203 kb)

## Abbreviations

circRNA: circular RNA
E: embryonic day
FPKM: fragments per kilobase of exon per million fragments mapped
KEGG: Kyoto Encyclopedia of Genes and Genomes
PCR: polymerase chain reaction
RPM: reads per million raw reads
TGF: transforming growth factor

## References

[1] Capel B, Swain A, Nicolis S, Hacker A, Walter M, Koopman P (1993). Circular transcripts of the testis-determining gene Sry in adult mouse testis. Cell..

[2] Hansen TB, Jensen TI, Clausen BH, Bramsen JB, Finsen B, Damgaard CK (2013). Natural RNA circles function as efficient microRNA sponges. Nature..

[3] Hansen TB, Kjems J, Damgaard CK (2013). Circular RNA and miR-7 in cancer. Cancer Res..

[4] Memczak S, Jens M, Elefsinioti A, Torti F, Krueger J, Rybak A (2013). Circular RNAs are a large class of animal RNAs with regulatory potency. Nature..

[5] Westholm JO, Miura P, Olson S, Shenker S, Joseph B, Sanfilippo P (2014). Genome-wide analysis of Drosophila circular RNAs reveals their structural and sequence properties and age-dependent neural accumulation. Cell Rep..

[6] Dehay C, Kennedy H (2007). Cell-cycle control and cortical development. Nat Rev Neurosci..

[7] Fish JL, Dehay C, Kennedy H, Huttner WB (2008). Making bigger brains-the evolution of neural-progenitor-cell division. J Cell Sci..

[8] Reiner O (2013). LIS1 and DCX: implications for brain development and human disease in relation to microtubules. Scientifica (Cairo).

[9] Sauleau P, Lapouble E, Val-Laillet D, Malbert CH (2009). The pig model in brain imaging and neurosurgery. Animal..

[10] Prather RS (2013). Pig genomics for biomedicine. Nat Biotechnol..

[11] Groenen MA, Archibald AL, Uenishi H, Tuggle CK, Takeuchi Y, Rothschild MF (2012). Analyses of pig genomes provide insight into porcine demography and evolution. Nature..

[12] Sauvageau M, Goff LA, Lodato S, Bonev B, Groff AF, Gerhardinger C (2013). Multiple knockout mouse models reveal lincRNAs are required for life and brain development. Elife..

[13] Sun E, Shi Y (2014). MicroRNAs: Small molecules with big roles in neurodevelopment and diseases. Exp Neurol..

[14] Ashwal-Fluss R, Meyer M, Pamudurti NR, Ivanov A, Bartok O, Hanan M (2014). circRNA biogenesis competes with pre-mRNA splicing. Mol Cell.

[15] Jeck WR, Sorrentino JA, Wang K, Slevin MK, Burd CE, Liu J (2013). Circular RNAs are abundant, conserved, and associated with ALU repeats. RNA..

[16] Liang D, Wilusz JE (2014). Short intronic repeat sequences facilitate circular RNA production. Genes Dev..

[17] Zhang XO, Wang HB, Zhang Y, Lu X, Chen LL, Yang L (2014). Complementary sequence-mediated exon circularization. Cell..

[18] Starke S, Jost I, Rossbach O, Schneider T, Schreiner S, Hung LH (2015). Exon circularization requires canonical splice signals. Cell Rep..

[19] Ivanov A, Memczak S, Wyler E, Torti F, Porath HT, Orejuela MR (2015). Analysis of intron sequences reveals hallmarks of circular RNA biogenesis in animals. Cell Rep..

[20] Kent WJ (2002). BLAT--the BLAST-like alignment tool. Genome Res..

[21] Huang R, Jaritz M, Guenzl P, Vlatkovic I, Sommer A, Tamir IM (2011). An RNA-Seq strategy to detect the complete coding and non-coding transcriptome including full-length imprinted macro ncRNAs. PLoS One..

[22] Vivancos AP, Guell M, Dohm JC, Serrano L, Himmelbauer H (2010). Strand-specific deep sequencing of the transcriptome. Genome Res..

[23] Salzman J, Chen RE, Olsen MN, Wang PL, Brown PO (2013). Cell-type specific features of circular RNA expression. PLoS Genet..

[24] Glazar P, Papavasileiou P, Rajewsky N (2014). circBase: a database for circular RNAs. RNA.

[25] Hinrichs AS, Karolchik D, Baertsch R, Barber GP, Bejerano G, Clawson H (2006). The UCSC Genome Browser Database: update 2006. Nucleic Acids Res..

[26] Hansen TB, Wiklund ED, Bramsen JB, Villadsen SB, Statham AL, Clark SJ (2011). miRNA-dependent gene silencing involving Ago2-mediated cleavage of a circular antisense RNA. EMBO J.

[27] Rybak-Wolf A, Stottmeister C, Glazar P, Jens M, Pino N, Giusti S (2015). Circular RNAs in the mammalian brain are highly abundant, conserved, and dynamically expressed. Mol Cell..

[28] Stipursky J, Francis D, Dezonne RS, de Araujo APB, Souza L, Moraes CA (2014). TGF-beta1 promotes cerebral cortex radial glia-astrocyte differentiation in vivo. Front Cell Neurosci..

[29] Rosso SB, Inestrosa NC (2013). WNT signaling in neuronal maturation and synaptogenesis. Front Cell Neurosci..

[30] Salinas PC (2012). Wnt signaling in the vertebrate central nervous system: from axon guidance to synaptic function. Cold Spring Harb Perspect Biol..

[31] Yi JJ, Barnes AP, Hand R, Polleux F, Ehlers MD (2010). TGF-beta signaling specifies axons during brain development. Cell..

[32] Nielsen KB, Kruhoffer M, Holm IE, Jorgensen AL, Nielsen AL (2010). 1Identification of genes differentially expressed in the embryonic pig cerebral cortex before and after appearance of gyration. BMC Res Notes..

[33] Tomoda T, Shirasawa T, Yahagi YI, Ishii K, Takagi H, Furiya Y (1996). Transforming growth factor-beta is a survival factor for neonate cortical neurons: coincident expression of type I receptors in developing cerebral cortices. Dev Biol..

[34] Hagelkruys A, Lagger S, Krahmer J, Leopoldi A, Artaker M, Pusch O (2014). A single allele of Hdac2 but not Hdac1 is sufficient for normal mouse brain development in the absence of its paralog. Development..

[35] Foti SB, Chou A, Moll AD, Roskams AJ (2013). HDAC inhibitors dysregulate neural stem cell activity in the postnatal mouse brain. Int J Dev Neurosci..

[36] Akizu N, Silhavy JL, Rosti RO, Scott E, Fenstermaker AG, Schroth J (2014). Mutations in CSPP1 lead to classical Joubert syndrome. Am J Hum Genet..

[37] Kaeser PS, Deng L, Wang Y, Dulubova I, Liu X, Rizo J (2011). RIM proteins tether Ca2+ channels to presynaptic active zones via a direct PDZ-domain interaction. Cell..

[38] Guan JS, Haggarty SJ, Giacometti E, Dannenberg JH, Joseph N, Gao J (2009). HDAC2 negatively regulates memory formation and synaptic plasticity. Nature..

[39] Schoch S, Mittelstaedt T, Kaeser PS, Padgett D, Feldmann N, Chevaleyre V (2006). Redundant functions of RIM1alpha and RIM2alpha in Ca(2+)-triggered neurotransmitter release. EMBO J..

[40] Kim D, Pertea G, Trapnell C, Pimentel H, Kelley R, Salzberg SL (2013). TopHat2: accurate alignment of transcriptomes in the presence of insertions, deletions and gene fusions. Genome Biol..

[41] Trapnell C, Williams BA, Pertea G, Mortazavi A, Kwan G, van Baren MJ (2010). Transcript assembly and quantification by RNA-Seq reveals unannotated transcripts and isoform switching during cell differentiation. Nat Biotechnol..

[42] Robinson JT, Thorvaldsdottir H, Winckler W, Guttman M, Lander ES, Getz G (2011). Integrative genomics viewer. Nat Biotechnol..

[43] Quinlan AR, Hall IM (2010). BEDTools: a flexible suite of utilities for comparing genomic features. Bioinformatics..

[44] Human H1 ESC cell line and differentiated SK-N-SH cell line data. http://hgdownload.cse.ucsc.edu/goldenPath/hg19/encodeDCC/wgEncodeCshlLongRnaSeq/.

[45] Kent WJ, Sugnet CW, Furey TS, Roskin KM, Pringle TH, Zahler AM (2002). The human genome browser at UCSC. Genome Res..

[46] Saeed AI, Sharov V, White J, Li J, Liang W, Bhagabati N (2003). TM4: a free, open-source system for microarray data management and analysis. Biotechniques..

[47] da Huang W, Sherman BT, Lempicki RA (2009). Bioinformatics enrichment tools: paths toward the comprehensive functional analysis of large gene lists. Nucleic Acids Res..

[48] da Huang W, Sherman BT, Lempicki RA (2009). Systematic and integrative analysis of large gene lists using DAVID bioinformatics resources. Nat Protoc..

[49] Clausen B, Fenger C, Finsen B (2013). In situ hybridization of cytokine mRNA using alkaline phosphatase-labelled oligodeoxynucleotide probes. Methods Mol Biol..

[50] Clausen BH, Lambertsen KL, Meldgaard M, Finsen B (2005). A quantitative in situ hybridization and polymerase chain reaction study of microglial-macrophage expression of interleukin-1beta mRNA following permanent middle cerebral artery occlusion in mice. Neuroscience..

