# Supplementary figures and images for: Spatio-temporal regulation of circular RNA expression during porcine embryonic brain development
Source: Genome Biol. 2015 Nov 5;16:245. doi: 10.1186/s13059-015-0801-3 (PMC4635978; doi:10.1186/s13059-015-0801-3)

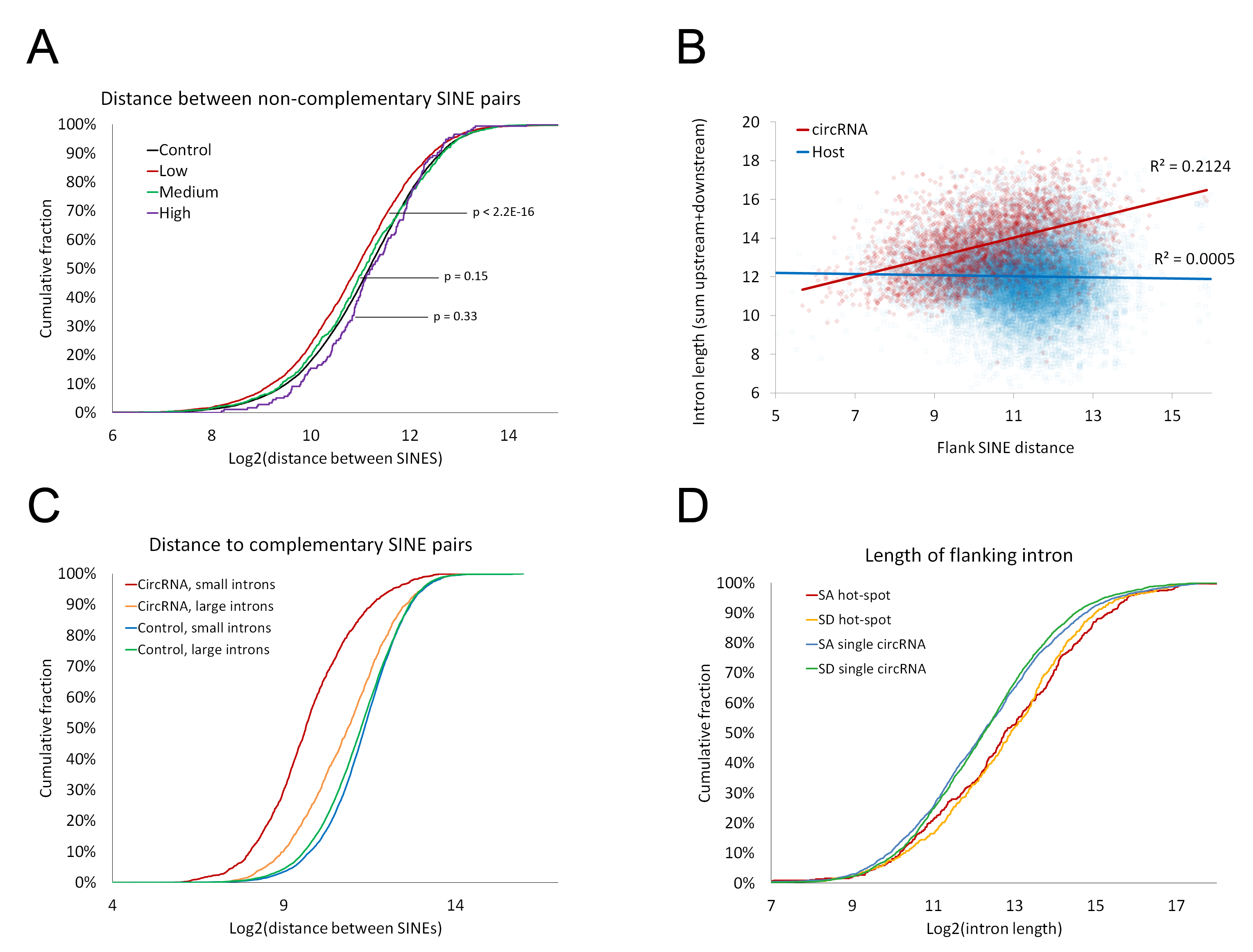

Supplement: Additional file 2: — Features of cortex circRNAs. a A cumulative plot of the distance between non-complementary SINE pairs in introns flanking circRNAs with expression levels categorized as either low (0.05–0.5 RPM, red line), medium (0.5 to 2.5 RPM, green line) or high (>2.5 RPM, purple line). Introns flanking exclusively linear spliced control exons (non-circRNA forming internal exons from genes that do form circRNAs at other exons) are shown with a black line. The figure is related to Fig. 1c. b Linear regression shows a positive correlation between intron length and distance between complementary intronic SINEs for circRNAs (red). This is not observed for non-circRNA flanking introns (blue). c Cumulative plot of the distance between complementary SINE pairs for circRNA flanking introns divided into the 50 % shortest introns and the 50 % longest introns (red and orange, respectively). Short circRNA flanking introns contain more proximal complementary SINEs. This is not observed for introns flanking control exons (blue and green). d Cumulative plot of flanking intron length for hot-spot exons producing two or more circRNAs from either splice acceptor (red) or splice donor (orange), and exons producing only one circRNA as either splice acceptor (blue) or splice donor (green). (TIFF 983 kb) [file 13059_2015_801_MOESM2_ESM.tif]

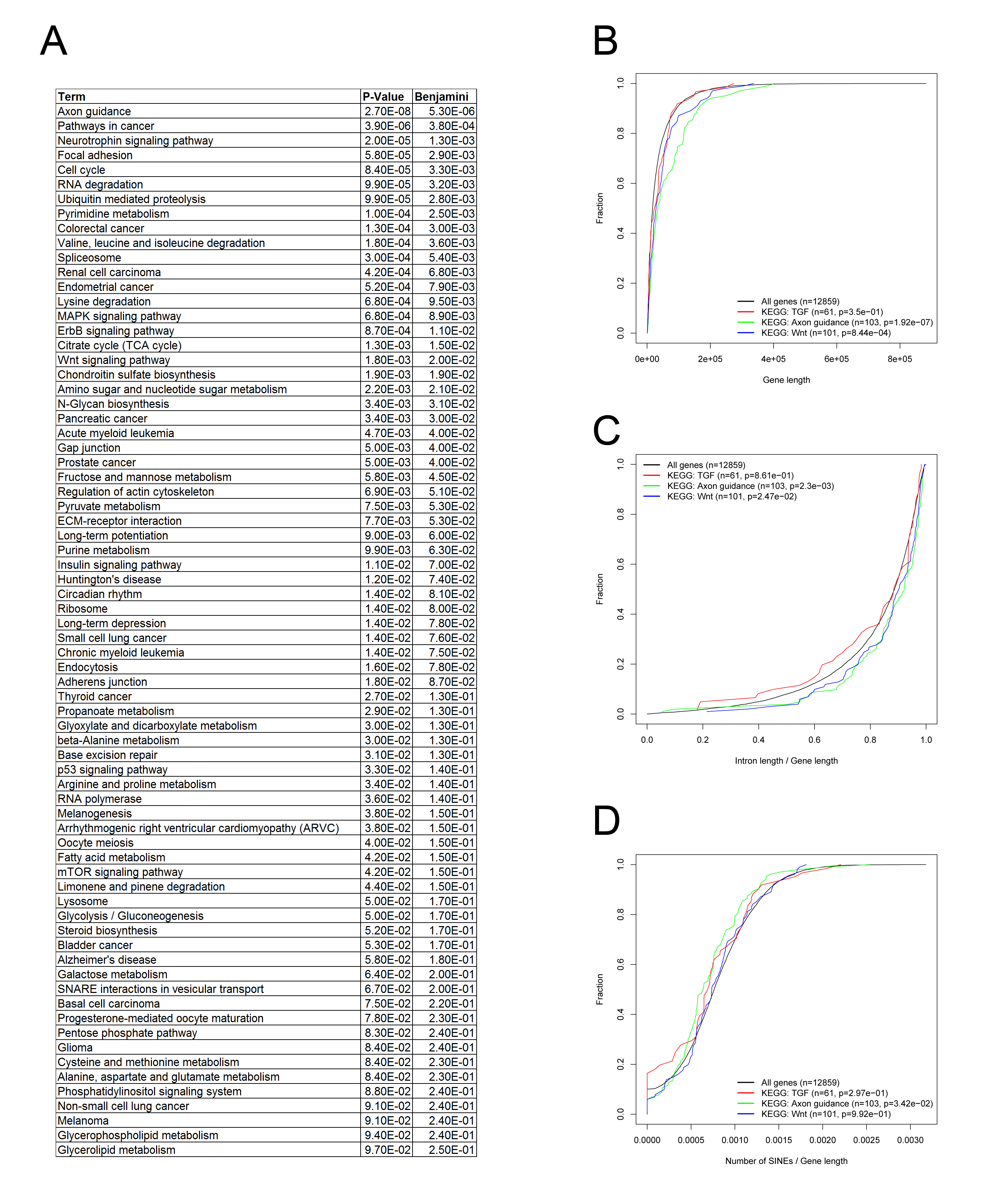

Supplement: Additional file 3: — Pathway analysis on highly expressed mRNAs and general pathway features. a DAVID pathway analysis was performed on the highest expressed mRNAs in embryonic cortex to find enriched KEGG pathways. mRNAs with expression above 50 FPKM were used for pathway analysis using the same background list as used for pathway analysis of circRNA host gene shown in Table 2. b–d Genes with two or more exons that are associated with the Wnt signaling pathway, axon guidance and the TGF-beta signaling pathway are examined relative to all genes for gene length (b), the proportion of intronic sequence in genes (c) and number of SINEs relative to gene length (d). Statistical significance for cumulative plots was calculated using the Wilcoxon test. (TIFF 619 kb) [file 13059_2015_801_MOESM3_ESM.tif]

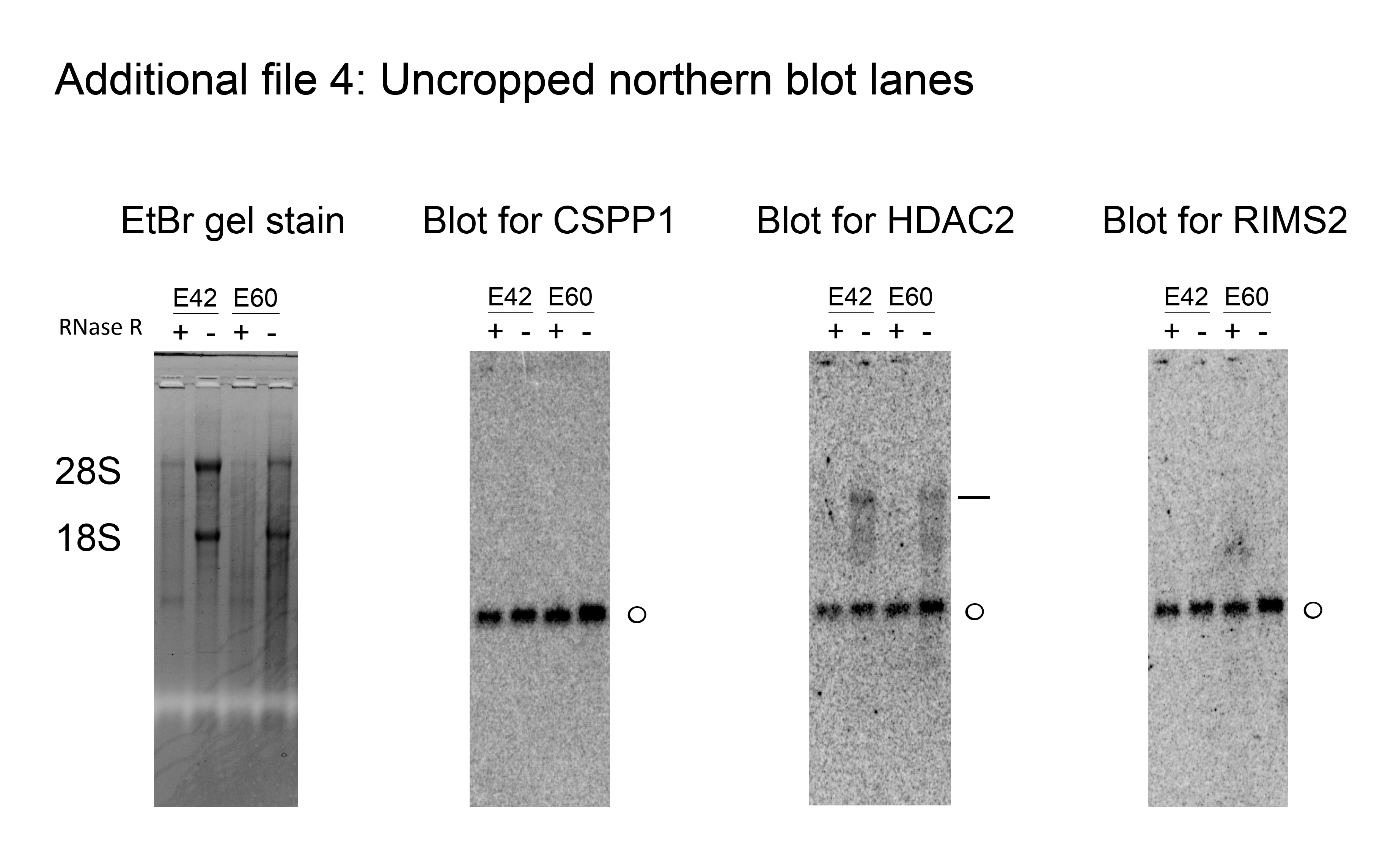

Supplement: Additional file 4: — Uncropped northern blot lanes. Northern blot lanes for circRNAs depicted in Fig. 3d are shown uncropped. Ethidium bromide stain of the gel prior to northern blot is also shown. (TIFF 3505 kb) [file 13059_2015_801_MOESM4_ESM.tif]
