# Supplementary material for: Spatio-temporal regulation of circular RNA expression during porcine embryonic brain development
Source: Genome Biol. 2015 Nov 5;16:245. doi: 10.1186/s13059-015-0801-3 (PMC4635978; doi:10.1186/s13059-015-0801-3)
Supplement: Additional file 5: — RT-PCR validation of hot-spot circRNA isoforms. RT-PCR with divergent primers showing multiple circRNA isoforms for the host genes TMEFF1, NDFIP2 and TLK1. Gel images show the sizes of back-spliced amplicons from RT-PCR. On the right, the expected size of circRNAs from RNA-seq is shown, with font size indicating general expression level. (PDF 203 kb) [file 13059_2015_801_MOESM5_ESM.pdf]

Cortex

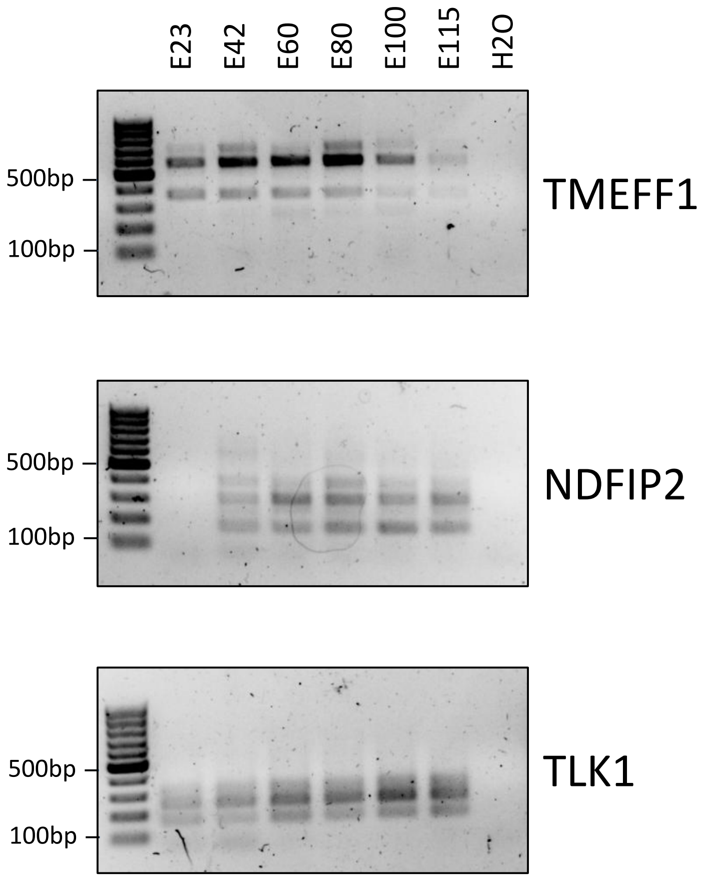

Expected/Predicted circRNA sizes from RNA-seq (Font size reflects isoform abundance based on RPM values):

703 bp
579 bp
364 bp
267 bp

586 bp
519 bp
394 bp
300 bp
166 bp

1097 bp
1030 bp
593 bp
337 bp (uses unannotated exon)
267 bp
191 bp
